# Unravelling the Genetics of Ischaemic Stroke

**DOI:** 10.1371/journal.pmed.1000225

**Published:** 2010-03-02

**Authors:** Hugh S. Markus

**Affiliations:** St. George's University of London, London, United Kingdom

## Abstract

Hugh Markus discusses genetic factors in stroke risk, and emphasizes the importance of large sample studies and rigorous replication of results in genetic stroke research.

**Summary Points**

- Epidemiological studies suggest genetic predisposition is important in stroke risk.
- Monogenic conditions are important for the individual patient but do not account for much population attributable risk. An increasing number of such conditions are being described, particularly for the small vessel disease stroke subtype.
- The candidate genes approach has proved disappointing in identifying genes contributing to the risk of multifactorial or polygenic stroke. This is a situation shared with other complex diseases.
- Recently the GWAS (genome-wide association study) approach has identified genetic loci for many other cardiovascular diseases such as coronary heart disease, diabetes, and hypertension, and is just being applied to stroke. Some novel genetic variants initially associated with other cardiovascular diseases have recently been identified as risk factors in stroke populations.

## Are Genetic Factors Important in Stroke Risk?

Stroke is the third commonest cause of death and the major cause of adult neurological disability. It is a major health problem not only in the developed world, but is increasing in incidence in much of the developing world. Cerebrovascular disease is also an important cause of dementia and age-related cognitive decline, and the commonest cause of late-onset epilepsy. Conventional risk factors such as high blood pressure account for a significant proportion of stroke risk but much risk remains unexplained, and we do not understand why some individuals with risk factors such as hypertension develop stroke while others with similar risk factor profiles remain disease free. It has been suggested that genetic factors may be responsible for some of this unexplained risk, but what is the evidence for this?

Limited data from twin studies suggests stroke is more common in monozygotic compared with dizygotic twins, consistent with a role for genetic factors [1]. Considerably more data is available for family history studies, which show a family history of stroke is more common in individuals with ischaemic stroke [1]. The association is stronger for younger individuals, and those with the large artery disease and small vessel disease subtypes of stroke [2],[3]. This association may represent genetic predisposition, but could also be explained by shared environmental factors. More robust data come from studying intermediate phenotypes of stroke. These are markers of disease, usually detected on imaging, which represent intermediate stages of disease pathology leading to stroke. Both twin and family studies have shown that MRI white matter hyperintensities, which usually represent small vessel disease [4], are the most heritable cerebrovascular phenotype, with a heritability (proportion of variation explained by genetic factors) estimated to be between 55% and 71% [5]–[7]. Carotid artery intima-media thickness, measured by ultrasound and believed to represent early stages of atherosclerosis and therefore relate to large artery stroke [8],[9], has been estimated to have a heritability of 30% to 68% [10]–[13].

## Why Should We Identify Genes for Stroke?

A small proportion of ischaemic stroke is monogenic [14]. A mutation in a specific gene results in disease, and most individuals with the abnormality are likely to develop stroke at some stage in their life. For these diseases identifying the underlying mutation allows diagnosis, prognostic information, specific treatments in some cases, and enables counselling of other family members. However, the vast majority of stroke appears to be “polygenic”; multiple genes, each likely to confer a small risk, interact with environmental risk factors to result in disease [15]. Identifying these underlying genetic risk factors may allow improved risk profiling, although as each genetic variant is likely to confer only a small risk, any useful risk prediction is likely to require a combination of multiple markers. Genetic testing for other polygenic diseases has already been developed, and indeed some gene tests for stroke are already marketed. However, their clinical relevance has been debated; most panels of genetic markers available explain only a small portion of total “genetic risk” [16]. Furthermore, their use has been questioned when clinicians already have difficulty treating known risk factors such as hypertension, which make a stronger contribution to risk, and concern has been expressed over the psychological consequences of testing. Perhaps more importantly, discovering genetic variants conferring increased stroke risk may allow new pathways involved in the pathogenesis of stroke to be identified and new treatments to be developed. This approach is just beginning to bear fruit in other “complex” diseases involving interactions between multiple genes and environment, such as macular degeneration and Crohn's disease [17],[18].

## How Can We Identify Genes for Stroke?

Linkage techniques have been successfully used to identify a number of genes causing monogenic stroke. This relies on identifying associations between chromosomal markers and disease phenotype within families. Linkage is good at identifying genes conferring greatly increased risk, but has been less successful in common polygenic complex disease such as stroke. There was excitement when this approach identified the *STRK1* gene in the Icelandic population as phosphodiesterase 4D [19], but this could not be replicated in other European populations [20].

Until recently the mainstay of investigating stroke genetics was the candidate gene method. Genetic variants (usually single nucleotide polymorphisms [SNPs]) are identified in a “candidate” gene that is hypothesised to be involved in stroke risk. The frequency of the SNP is compared in a group of stroke patients compared with controls. Many hundreds of such studies have been carried out in stroke with largely disappointing results [21]. This picture is common to many other complex diseases, and the reasons for lack of success have been explored in detail [21]. Important factors include small sample sizes, failure to phenotype cases accurately, and failure to replicate positive associations, combined with publication bias resulting in preferential publication of positive associations. A major problem with the candidate gene method is that associations can be identified only in genes already discovered and implicated in the disease. Completely novel genes involved in disease risk cannot be identified.

### The Genome-Wide Association Study (GWAS) Approach

**Figure 1:**
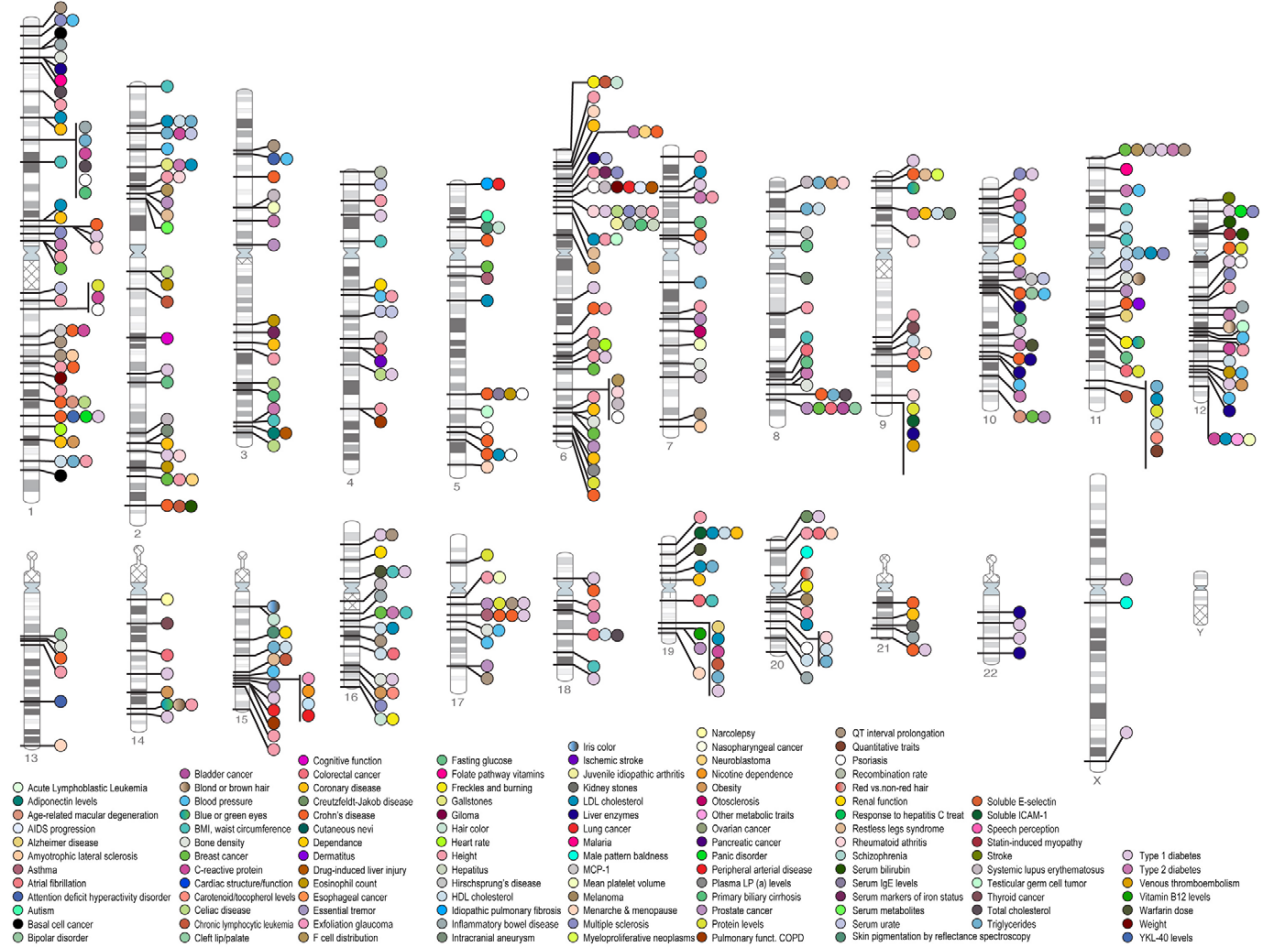
Published associations between chromosomal loci and many diseases which have been identified by genome-wide associations up to June 2009. A significance level of *p*<5×10^−8^ has been used to determine whether associations are significant. As can be seen a large number of associations have been identified with many common diseases. *Credit: Darryl Leja and Teri Manolio; available from* http://www.genome.gov/gwastudies. *Accessed 25 September 2009*.

Recently the field of complex genetics has been revolutionised by the genome-wide association study (GWAS) method [22]. This allows up to 1 million SNPs spanning the whole genome to be genotyped in a single individual. Using a case control methodology, and rigorous statistical methods to account for the multiple comparisons made, associations between completely unexpected chromosomal loci and disease can be identified. With this approach, combined with much larger study sizes involving thousands of patients and rigorous replication of positive results, over 600 new genetic loci have been discovered predisposing to other complex disorders such as diabetes and myocardial infarction (Figure 1) [23]. An early success was in age-related macular degeneration, which, like stroke, is primarily a disease of the elderly in which cardiovascular risk factors increase risk [24]. Robust and replicable associations were identified with complement factor H, and this has allowed new treatment approaches to be developed. Novel genetic association have now been reported in many cardiovascular diseases including myocardial infarction, diabetes, obesity, hypertension, and hyperlipidaemia [23]. An important message from these studies is that most genetic variants discovered using this approach individually account for only a small increase in risk, with odds ratios usually between 1.1 and 1.3. This means large sample sizes are required to identify such variants.

### The GWAS Approach in Stroke

Stroke genetics has lagged behind that of many other complex diseases, and the GWAS approach is only just beginning to be applied. An early study in 500 individuals found no definite associations, but we now appreciate this was underpowered [25]. Larger studies are currently taking place in the UK (as part of the Wellcome Case Control Consortium 2), the US, Australia, and other countries. As yet most data, and most current studies, are from white populations. Other complex diseases have taught us that we may need very large sample sizes to identify genetic variants. For example, in hypertension, initial studies in a few thousand cases were disappointing. More recently meta-analysis of over 80,000 cases identified eight novel genetic loci [26]. The importance of robust replication prior to publication was demonstrated by the recent high-profile publication of a novel Chromosome 12 locus associated with stroke identified on GWAS [27], which unfortunately could not be replicated in a sample of over 10,000 individuals, although differences in study designs—the original study was based on prospective cohorts and the replication was cross-sectional—could potentially account for some of the discrepancy [28]. The lack of success in stroke so far is likely to reflect the relatively small sample sizes studied to date, but other issues may also be important. For example, the GWAS approach relies on the existence of common variants, each of which confer risk. It is less good at detecting rare variants which could also confer increased risk.

Some novel genetic variants initially associated with other cardiovascular diseases have recently been identified as risk factors in stroke populations. A Chromosome 9 variant associated with myocardial infarction and coronary artery disease [29] was found to also be associated with stroke across multiple populations, but this association was due to an association with large artery stroke, and no association was found with other stroke subtypes [30]. Two other variants identified as risk factors for atrial fibrillation have also been associated with stroke; here the association was primarily with cardioembolic stroke [31],[32]. These findings emphasise that genetic risk factors may predispose to specific subtypes of stroke. Therefore, identifying such risk factors will depend on rigorous stroke phenotyping and large numbers of cases with each stroke subtype.

## Monogenic Stroke Disorders

A large number of rare monogenic disorders can cause stroke (Table 1) Some of these result in stroke as part of a systemic disorder, while others produce a clinical phenotype limited to the central nervous system [14]. Progress has been particularly exciting in cerebral small vessel disease. This causes lacunar stroke, which accounts for about 20% of ischaemic stroke, and is the most common cause of vascular dementia. The most common monogenic form of small vessel disease is CADASIL (cerebral autosomal dominant arteriopathy with subcortical infarcts and leukoencephalopathy), which results from mutations in the *NOTCH3* gene [33]. A striking feature of CADASIL is that, even within families, disease severity is highly variable. This variation is not accounted for by the site of the disease mutation. Confluent white matter hyperintensity called leukoaraiosis, and best seen on MRI, is a prominent feature in CADASIL. A recent study measuring leukoaraiosis volume demonstrated that the variability in leukoariosis volume between different CADASIL carriers has a strong genetic predisposition, with a heritability as high as 63% [34]. This raises the intriguing possibility that other genes modulate the damage caused by the *NOTCH3* mutation to modify the disease phenotype. GWAS studies are currently being planned to try to identify these modifying genes. Whether such genes modify only leukoaraiosis severity in CADASIL, or whether they could also play an important role in modifying white matter damage in response to other more common risk factors such as hypertension, remains to be determined. Similar GWAS studies are being carried out using the intermediate phenotype of white matter hyperintensity lesion volume both in normal populations and in populations with stroke and stroke risk factors. It will be interesting to compare the results of the studies in different populations.

**Table 1:** Monogenic or single gene disorders causing stroke, classified according to the each one's stroke subtype.

Stroke Subtype	Specific Monogenic Disease
Small vessel disease	CADASIL
	CARASIL
	Cerebrovascular retinopathy and HERNS
	COL4A1 small vessel arteriopathy with haemorrhage
Large artery atherosclerosis and other arteriopathies	Familial hyperlipidaemias
	Moya-Moya disease
	Pseudoxanthoma elasticum
	Neurofibromatosis type I
Large artery disease—dissection	Ehlers-Danlos syndrome type IV
	Marfan syndrome
	Fibromuscular dysplasia
Disorders affecting both small and large arteries	Fabry disease
	Homocysteinuria
	Sickle cell disease
Cardioembolism	Familial cardiomyopathies
	Familial arrhythmias
	Hereditary haemorrhagic telangiectasia
Prothrombotic disorders	
Mitochondrial disorders	MELAS

HERNS, hereditary endotheliopathy with retinopathy, nephropathy, and stroke; MELAS, Mitochondrial myopathy, encephalopathy, lactic acidosis, and stroke.

Genes causing a number of rarer monogenic forms of small vessel disease have recently been identified. CARASIL (cerebral autosomal recessive arteriopathy with subcortical infarcts and leukoencephalopathy), which causes lacunar stroke, leukoaraiosis, and early onset vascular dementia, has been shown to results from mutations in the HtrA serine protease 1 (*HTRA1*) gene, which is involved in TGF-β signalling [35]. Autosomal dominant retinal vasculopathy with cerebral leukodystrophy is a microvascular endotheliopathy presenting with visual loss, stroke, and dementia with onset in middle age. C-terminal frameshift mutations in *TREX1*, which encodes a DNA-specific 3′ to 5′ exonuclease ubiquitously expressed in mammalian cells were identified [36]. These truncated proteins retain exonuclease activity but lose normal perinuclear localization. *COL4A1*, a gene encoding the type IV collagen alpha1 chain, has been found to be involved in families with autosomal-dominant porencephaly and infantile hemiparesis [37]. Patients have been reported to present with adult-onset white matter ischaemic changes consistent with small vessel disease with microbleeds in the absence of infantile hemiparesis or intracerebral haemorrhage [38]. Fabry disease is an X-linked recessive lysosomal storage disease resulting from deficient alpha-galactosidase which causes organ failure in multiple beds including the brain. Stroke may result from a number of mechanisms including cerebral small vessel disease, and confluent white matter ischaemic changes are seen on MRI. It was reported be an important cause of young-onset cryptogenic ischemic stroke, accounting for 4.9% of male cases and 2.4% of female cases, although further studies are required to confirm this [39]. Therefore multiple different gene defects resulting in disruption of multiple different pathways can result in the a similar clinical phenotypes of cerebral small vessel disease.

## Conclusions

In summary, genetic factors appear to be important in stroke risk, although we do not really know how important. Candidate gene studies have been largely disappointing but with GWAS technology we may well be at the dawn of a new era in stroke genetics. Future technological advances such as copy number variant determination and, whole genome sequencing are also likely to be important; the latter will allow rare variants associated with disease to be identified. Studies to date have emphasised the importance of careful phenotyping or stroke subtyping, and the experience of other complex diseases has taught us that we need large sample studies and rigorous replication of results.

## Abbreviations

CADASIL: cerebral autosomal dominant arteriopathy with subcortical infarcts and leukoencephalopathy
CARASIL: cerebral autosomal recessive arteriopathy with subcortical infarcts and leukoencephalopathy
GWAS: genome-wide association study
SNP: single nucleotide polymorphism
